# Antifungal activity and mechanism of action of Ou-gon (*Scutellaria* root extract) components against pathogenic fungi

**DOI:** 10.1038/s41598-019-38916-w

**Published:** 2019-02-08

**Authors:** Xia Da, Yayoi Nishiyama, Duerna Tie, Kyaw Zaw Hein, Osamu Yamamoto, Eishin Morita

**Affiliations:** 1 Department of Dermatology, Faculty of Medicine, Shimane University, Izumo, 693-8501 Japan; 2 Institute of Medical Mycology, Teikyo University, Tokyo, 192-0395 Japan; 3 Robert and Arlene Kogod Center on Aging, Mayo Clinic, Rochester, MN 55905 USA; 4 Division of Dermatology, Department of Medicine of Sensory and Motor Organs, Faculty of Medicine, Tottori University, Yonago, 683-8503 Japan

## Abstract

Ou-gon, an extract from *Scutellaria baicalensis* Georgi root, has been shown to exhibit pronounced antifungal activity. The present study aimed to identify antifungal components of Ou-gon and to determine their mechanism of action against pathogenic fungi. Antifungal activity was assessed by the microbroth dilution method using four common human pathogenic fungi, *Trichophyton rubrum*, *Trichophyton mentagrophytes*, *Aspergillus fumigatus*, and *Candida albicans*. Components of crude Ou-gon extract were separated by reversed-phase high-performance liquid chromatography. Active antifungal components were identified by liquid chromatography-electrospray ionization tandem mass spectrometry. Terminal deoxynucleotidyl transferase dUTP nick end-labelling assay, SYTOX^®^ green uptake assay, determination of intracellular reactive oxygen species and mitochondrial membrane potential as well as microscopy (confocal laser microscopy, scanning and transmission electron microscopy) were used to probe the mode of action. Two components with potent antifungal activity, baicalein and wogonin, were identified in Ou-gon. Baicalein showed potent antifungal activity against the four fungi tested. Wogonin displayed antifungal activity against all four fungi except *C. albicans*. The components are considered to induce apoptosis-like programmed cell death via hyperproduction of reactive oxygen species. This study enhances our understanding of the antifungal activity of Kampo medicine, and may contribute to the development of new and safe antifungal therapeutics.

## Introduction

Fungal infections are potentially fatal complications associated with the use of popular immunosuppressive drugs, including anticancer drugs^1,2^, and hence, the development of safe antifungal drugs for clinical use would greatly benefit patients. The rise in the incidence of fungal infections has exacerbated the need for next-generation antifungal agents, as many of the currently available drugs have undesirable side effects, are ineffective against new or re-emerging fungal strains, or lead to rapid development of resistance^3–5^. Moreover, fungal infections can be dangerous for patients who receive anticancer or immunosuppressive agents, including immunocompromised patients, because immunosuppression renders the patient vulnerable to viral, bacterial, and fungal infections^6,7^. Therefore, the discovery of antifungal drugs with low toxicity, broad spectrum of activity, and a new mode of action is becoming increasingly important^6–8^.

Natural products provide the basis for the vast majority of anti-infective therapies currently in clinical use. For example, polyenes, echinocandins, and flavonoids, three key classes of antifungal drugs, are natural product derivatives^9,10^. Kampo is a well-known type of traditional Japanese medicine that is widely used to relieve symptoms of a variety of diseases because of the low incidence of adverse effects associated with its use. It is currently prescribed by over 80% of medical doctors in Japan^7^. Kampo medicines mainly contain crude extracts from natural products (plants, animals, and minerals) that are prepared according to classical Kampo methodologies. Because plants synthesise numerous antimicrobial components, e.g., defensins, Kampo medicines likely contain potent antimicrobial and antifungal compounds^11,12^.

We previously evaluated the antifungal activity of 61 commercially available Kampo medicines towards *Trichophyton rubrum* using the microbroth dilution assay^7^. Seven of these medicines exhibited antifungal activity, with six containing Ou-gon, an extract from the roots of *Scutellaria baicalensis* Georgi. Furthermore, crude Ou-gon extract exhibited pronounced antifungal activity^7^. Ou-gon is one of the popular crude drugs in Kampo medicine, traditionally used in the Far East because of its anti-inflammatory, antimicrobial, and anti-allergic activities^13,14^. The identification of the active constituents of Ou-gon would facilitate their synthesis and structural modification to obtain active components with enhanced efficacy and potential therapeutic usefulness. The current study aimed to identify antifungal components in Ou-gon and to determine their mechanism of action against pathogenic fungi.

## Results

### Antifungal activity of Ou-gon extracts

**Figure 1:**
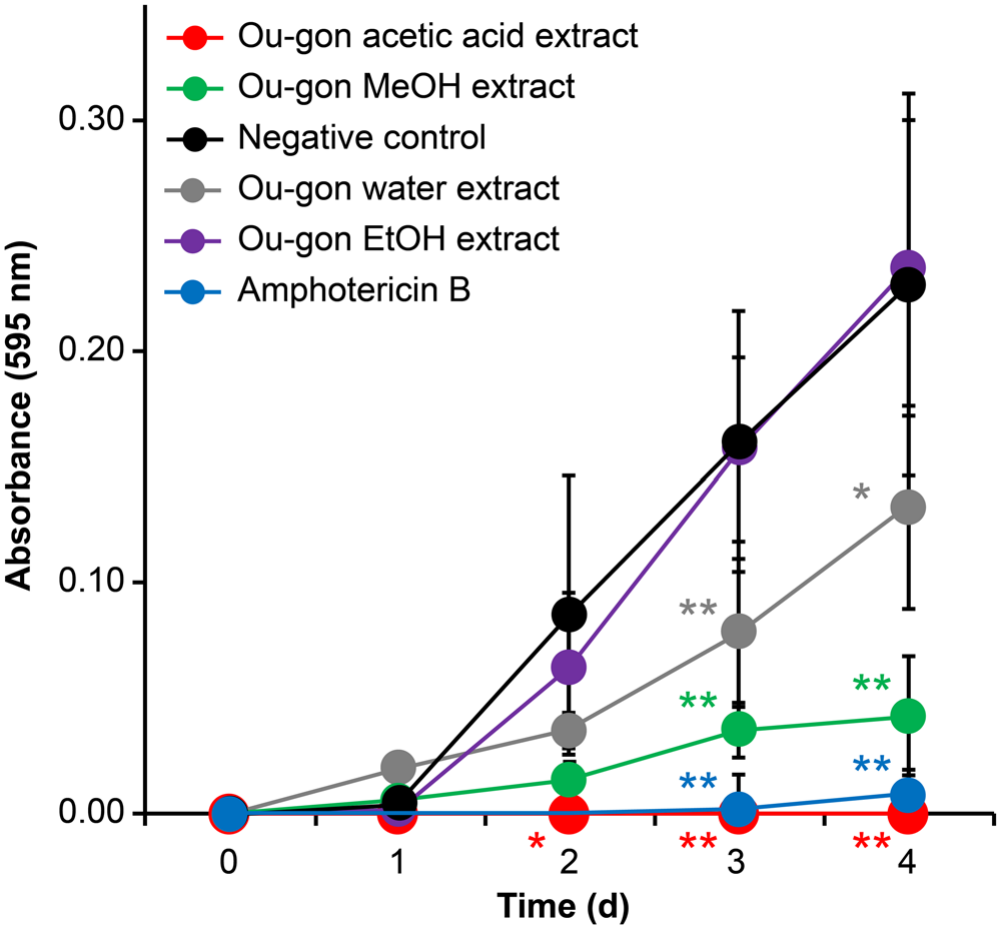
*In-vitro* antifungal activity of crude Ou-gon extracts (20 mg/mL) against *T. rubrum* in Sabouraud liquid medium at 28 °C from day 0 to 4. Data are presented as the mean ± SD of four independent experiments; *p < 0.05, **p < 0.01 compared with negative control (no treatment).

The antifungal activities of four Ou-gon extracts (water, MeOH, EtOH, and acetic acid extracts) were examined in a microbroth dilution assay using *T. rubrum*. The acetic acid, MeOH, and water extracts exhibited pronounced antifungal activity, as shown in Fig. 1, whereas the EtOH extract showed no activity. Among the three extracts that exhibited antifungal activity, the acetic acid extract displayed the strongest activity, comparable to that of amphotericin B, as shown in Fig. 1.

### Isolation and identification of the antifungal components of Ou-gon

**Figure 2:**
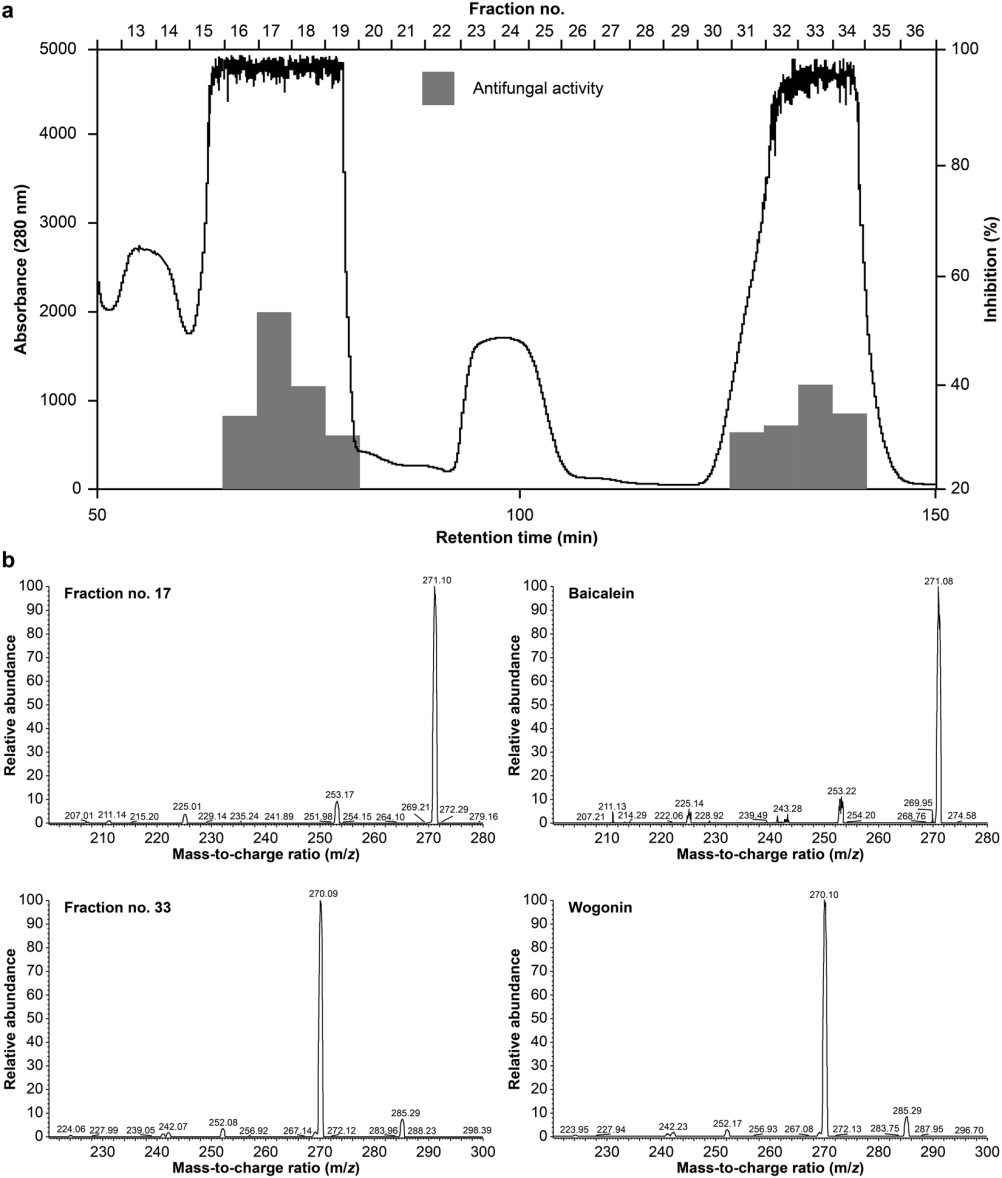
(**a**) C18 column-attached RP-HPLC profile of the Ou-gon acetic acid extract (sample amount: 160 mg; injection volume: 1 mL). The bars show the antifungal activity of each fraction, which was determined in a microbroth assay with *T. rubrum* in each experiment. (**b**) LC-ESI-MS/MS analysis of fractions no. 17 and 33, and compound standards. The samples were analysed in a positive ion mode, using a TSQ quantum mass spectrometer. The mass peaks were selected and m/*z* values calculated using Xcalibur 2.1 software.

Because of its most pronounced relative antifungal activity, the acetic acid extract was selected for reversed-phase high-performance liquid chromatography (RP-HPLC) analysis. The extract was separated by RP-HPLC on a C18 column, and the antifungal activity of the eluted fractions was tested. Fractions no. 16–19 and 31–34 exhibited antifungal activity, as shown in Fig. 2a.

When fraction no. 17 was analysed by liquid chromatography-electrospray ionization tandem mass spectrometry (LC-ESI-MS/MS), a strong peak, indicating high relative ion abundance, was detected at 271.10 (m/*z*). This ion represents baicalein, a major component of Ou-gon (Fig. 2b). Similarly, fraction no. 33 was identified to contain a relatively high amount of wogonin (Fig. 2b)^15^.

### Antifungal activity of baicalein and wogonin

**Figure 3:**
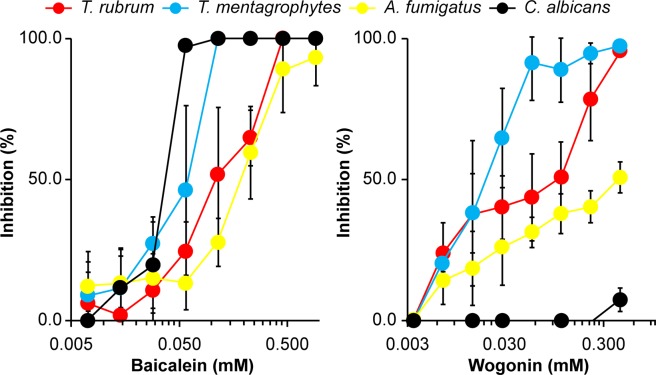
Antifungal activity of pure baicalein and wogonin against *T. rubrum*, *T. mentagrophytes*, *A. fumigatus*, and *C. albicans* on day 4 of culture. Data are presented as the mean ± SD from three independent experiments. The growth inhibition rate was calculated using the following formula^7^: growth inhibition rate (%) = [(absorbance_control_ – absorbance_treated_)/absorbance_control_] × 100.

Fungal culture in the presence of various concentrations of baicalein revealed pronounced concentration-dependent antifungal activity against *T. rubrum*, *Trichophyton mentagrophytes*, *Aspergillus fumigatus*, and *Candida albicans* (Fig. 3). Wogonin exhibited antifungal activity towards all fungi except *C. albicans* (Fig. 3). The minimal inhibitory concentrations (MIC_50_) of baicalein for *T. rubrum*, *T. mentagrophytes*, *A. fumigatus*, and *C. albicans* were 0.12 mM, 0.06 mM, 0.23 mM, and 0.03 mM, respectively. The MIC_50_ of wogonin for *T. rubrum*, *T. mentagrophytes*, and *A. fumigatus* were 0.06 mM, 0.03 mM, and 0.23 mM, respectively.

### Effect of baicalein and wogonin on cellular fungal functions

**Figure 4:**
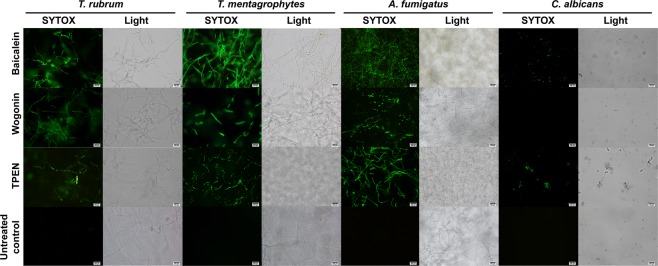
SYTOX^®^ green staining of fungal cells treated with baicalein (0.46 mM), wogonin (0.44 mM), or TPEN (2 μM) at 24 h after treatment. Light, light microscopy image. Scale bars, 500 μm. The images are representative of four SYTOX^®^ green uptake assay experiments.

Upon exposure to baicalein, *T. rubrum*, *T. mentagrophytes*, *A. fumigatus*, and *C. albicans* cells were stained with the SYTOX^®^ green nucleic acid stain (Fig. 4). Wogonin affected the cell membrane integrity of all fungi tested except *C. albicans*.

**Figure 5:**
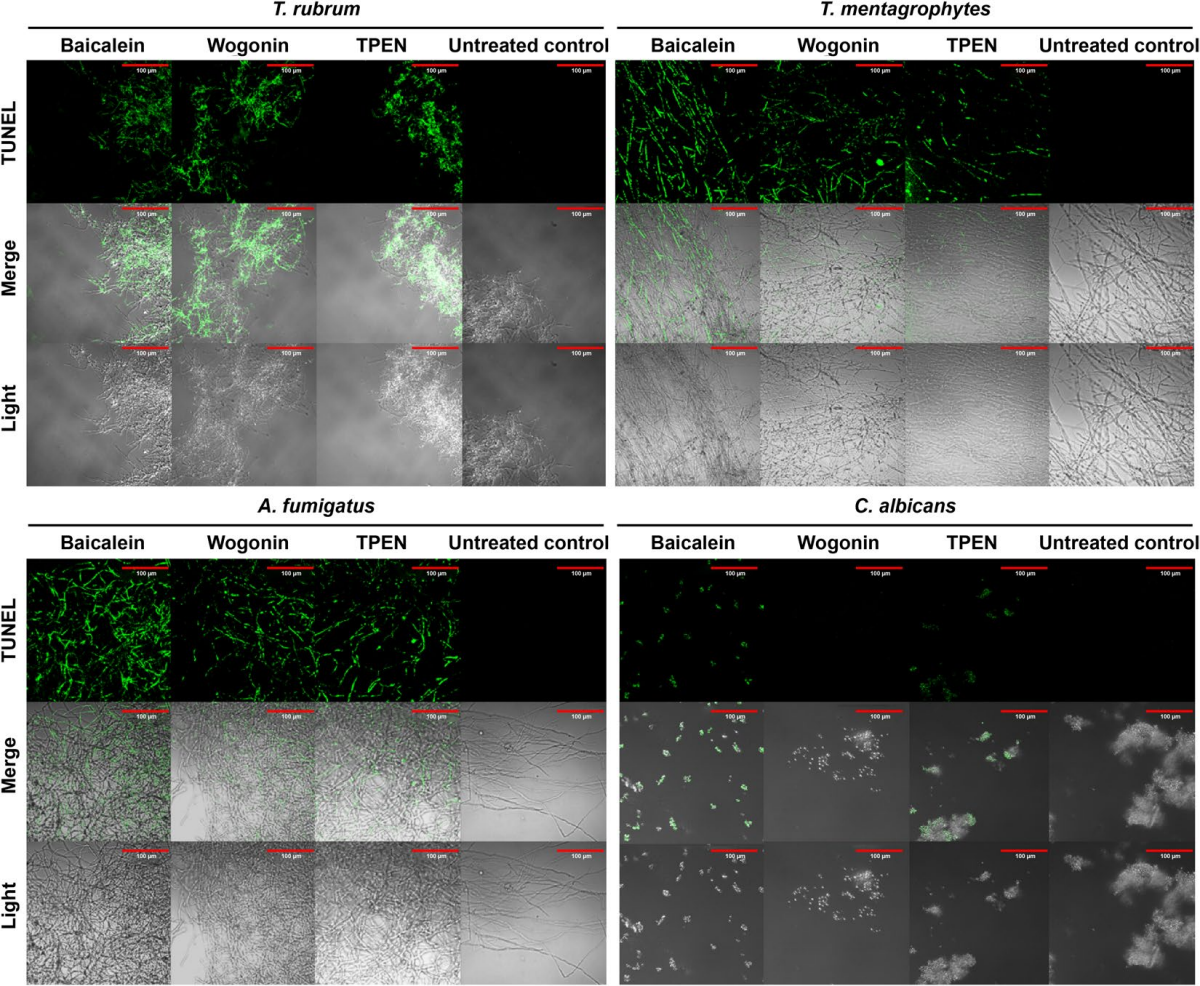
DNA damage in *T. rubrum*, *T. mentagrophytes*, *A. fumigatus*, and *C. albicans* cells induced by baicalein and wogonin, as determined by the TUNEL assay. Fungi were treated with baicalein (0.46 mM), wogonin (0.44 mM), or TPEN (2 μM) for 24 h. Light, light microscopy image. Scale bars, 100 μm. The images are representative of four TUNEL assay experiments.

Pronounced terminal deoxynucleotidyl transferase dUTP nick end-labelling (TUNEL) staining of baicalein-treated *T. rubrum*, *T. mentagrophytes*, *A. fumigatus*, and *C. albicans* cells was observed (Fig. 5). Wogonin induced TUNEL staining in all fungi tested except *C. albicans*.

**Figure 6:**
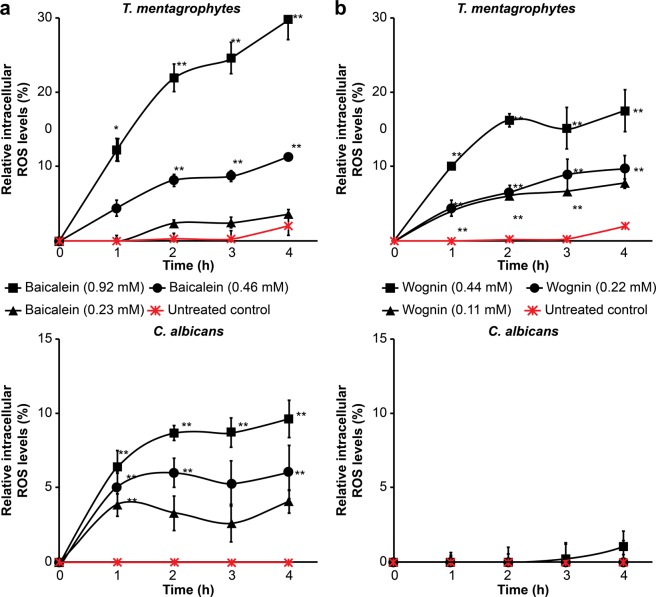
ROS generation in baicalein- and wogonin-treated *T. mentagrophytes* and *C. albicans* cells. Data are presented as the mean ± SD from three independent experiments; *p < 0.05, **p < 0.01 compared with a non-treated control. Fluorescence intensity was determined at 485-nm excitation wavelength and 535-nm emission wavelength. Relative intracellular ROS levels were calculated using the following formula^57^: relative intracellular ROS level = [(fluorescence intensity_measurement time_ – fluorescence intensity_initial time_)/fluorescence intensity_initial time_] × 100.

Reactive oxygen species (ROS) accumulation was evaluated in *T. mentagrophytes* and *C. albicans*. The fungal cells were examined using the fluorescent dye 2′, 7′-dichlorofluorescin diacetate (DCFDA) after incubation with baicalein and wogonin. Baicalein treatment induced concentration-dependent ROS accumulation in *T. mentagrophytes* and *C. albicans* (Fig. 6a), whereas wogonin treatment induced ROS accumulation only in *T. mentagrophytes* (Fig. 6b).

**Figure 7:**
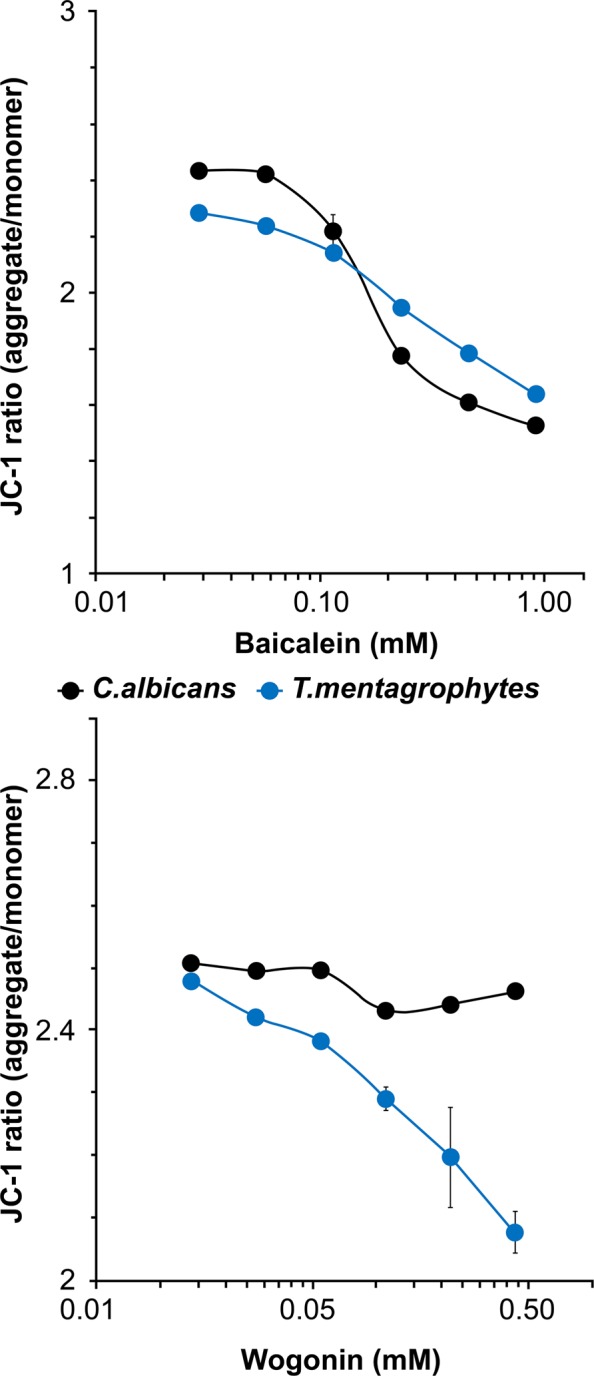
MMP assay of baicalein- or wogonin-treated *T. mentagrophytes* and *C. albicans* cells. Data are presented as the mean ± SD from three independent experiments. The excitation and emission wavelengths were 485 nm and 535 nm, respectively, for the green monomers, and 485 nm and 595 nm, respectively, for the red aggregates of JC-1.

Changes in the mitochondrial membrane potential (MMP) in *T. mentagrophytes* and *C. albicans* cells were evaluated using the specific fluorescent probe JC-1 after incubation with baicalein or wogonin. Baicalein treatment resulted in a concentration-dependent decrease in MMP in *T. mentagrophytes* and *C. albicans*, whereas wogonin treatment caused a decrease in MMP only in *T. mentagrophytes* (Fig. 7).

### Ultrastructural analysis of fungal cells

**Figure 8:**
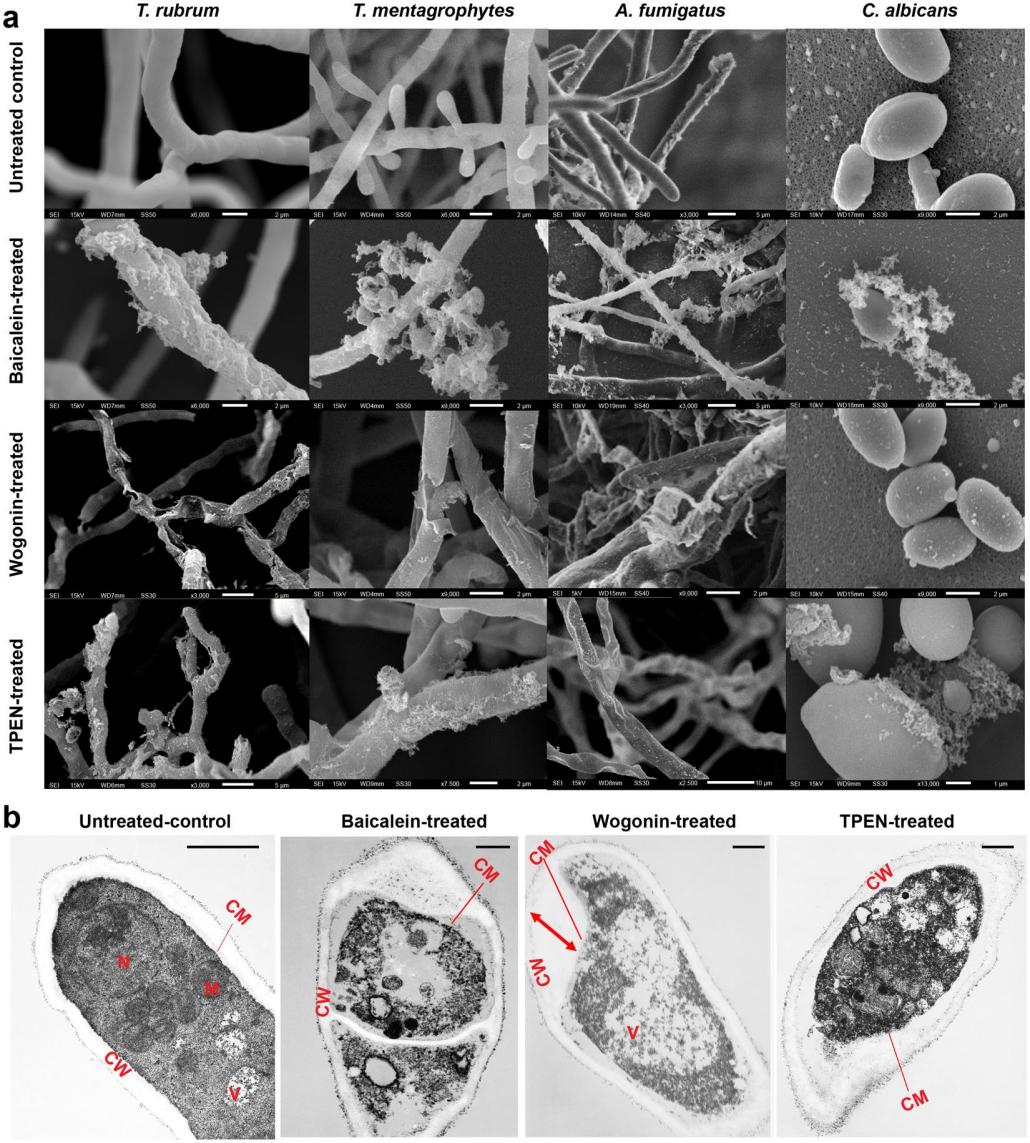
(**a**) SEM images of baicalein- (0.46 mM), wogonin- (0.44 mM), and TPEN- (2 μM) treated *T. rubrum*, *T. mentagrophytes*, *A. fumigatus*, and *C. albicans* cells for 24 h. The images shown are representative of SEM experiments. The red arrow represents the increased volume of the outer half layer of the cell wall. (**b**) TEM images of baicalein- (0.46 mM), wogonin- (0.44 mM), and TPEN- (2 μM) treated *T. rubrum* for 24 h. Ultrastructural alterations of the cell membrane and organelles are apparent in baicalein- and wogonin-treated hyphae. On the other hand, wogonin-treated hyphae show mainly cell wall changes. CM, cell membrane; CW, cell wall; M, mitochondria; N, nucleus; V, vacuole. Scale bars, 1 μm. The red arrow indicates the swelling of the fungal cell wall. The images are representative of four independent experiments.

As illustrated in Fig. 8a, non-treated fungal cells exhibited the expected filamentous forms, and no extracellular material was observed in scanning electron microscopy (SEM). Baicalein induced deformation of the fungal surface structure and efflux of a cotton-like substance, which was thought to be degenerated cytosol, around fungal bodies of *T. rubrum*, *T. mentagrophytes*, *A. fumigatus*, and *C. albicans*. Wogonin treatment caused shrinkage or cracking of fungal filaments in all fungi tested except *C. albicans* (Fig. 8a).

Transmission electron microscopy (TEM) of *T. rubrum* revealed clear differences in cytoplasmic organelles between non-treated and baicalein-treated hyphae. As shown in Fig. 8b, after 24 h incubation of *T. rubrum* in the presence of baicalein, the cytoplasmic membrane was disordered, the cell organelles were degenerated, amorphous areas were enlarged, and the cell membrane was detached from the cell wall. Wogonin treatment resulted in swollen cell walls, whereas the cytoplasm was largely intact (Fig. 8b).

## Discussion

The current study demonstrated that baicalein and wogonin, the major flavones in Ou-gon extracts^16^, possess potent antifungal activity against pathogenic fungi, as determined by the microbroth dilution assay. The SYTOX^®^ green uptake assay was used to detect cell damage, revealing structural disintegration of the plasma membrane^17^. Upon exposure to baicalein and wogonin, the clear SYTOX^®^ green staining of fungal cells indicated a loss of cell membrane integrity. The TUNEL assay was used to determine the induction of apoptotic DNA fragmentation, as described previously^17^. TUNEL staining of baicalein- and wogonin-treated fungal cells suggested the presence of apoptotic DNA breaks. Apoptosis as a mechanism of baicalein- and wogonin-induced cell death was supported by the finding that the fungal cells produced ROS upon exposure to these compounds. ROS accumulation is considered one of the primary biochemical causes of apoptosis. Inappropriate regulation of ROS levels can damage cells, leading to abnormal fungal growth and consequential apoptotic-like cell death^18,19^. Changes in the MMP, indicating the opening of transition pores in the mitochondrial membrane and release of apoptogenic factors into the cytosol, are considered another characteristic of apoptosis^20,21^. Intracellular ROS accumulation and MMP reduction can be regarded as the keys to the antifungal activity of baicalein and wogonin. Apoptosis-like programmed cell death thus likely constitutes the antifungal mechanism of baicalein and wogonin.

Further evidence of ultrastructural changes of fungal cells associated with baicalein and wogonin exposure was obtained from SEM and TEM analyses. The two compounds induced different ultrastructural changes. The degeneration of cytoplasmic organelles and efflux of cytosolic contents in baicalein-treated hyphae as indicated by SEM and TEM suggest that baicalein possibly induced disturbance of the plasma and intracytoplasmic membrane synthesis, thereby impairing membrane function and leading to morphological changes. The partial swelling, and shrinkage or cracking of the cell wall found in wogonin-treated fungal cells suggest that wogonin perturbs the biosynthesis of cell wall components, thus inhibiting cell wall synthesis. Similar ultrastructural changes in the cell wall have been observed *in vivo* in *T. rubrum* treated by luliconazole^22^, a novel topical imidazole that targets lanosterol 14-alpha-demethylase (CYP51A1), an enzyme involved in the biosynthesis of ergosterol, and the MIC_80_ value of luliconazole against *C. albicans* was more than 100 times higher than that against *T. rubrum*^23^.

Baicalein exhibited antifungal activity against *T. rubrum*, *T. mentagrophytes*, *A. fumigatus*, and *C. albicans*. Although the chemical structures of baicalein and wogonin are similar^24^, wogonin did not exhibit antifungal activity against *C. albicans* (Figs 4 and 5). Baicalein reportedly exhibits antifungal activity against *C. albicans*, *Candida tropicalis*, *Candida parapsilosis*, *Cryptococcus neoformans*, and *Pityrosporum ovale*^25–28^. Baicalein induces apoptosis in *C. albicans*, and the combination of baicalein and amphotericin B accelerates apoptosis in *C. albicans* cells, which is accompanied by increased intracellular ROS levels^27,29^. In addition, baicalein reduces the cell surface hydrophobicity of *C. albicans* biofilms by decreasing *CSH1* mRNA expression^26^. Further, a combination of baicalein and fluconazole showed strong antifungal activity against fluconazole-resistant *C. albicans*, which was accompanied by inhibition of the efflux pumps of *C. albicans*^30^. In the current study, the MIC_50_ value of baicalein for *C. albicans* was determined as 0.03 mM, which was consistent with a previous study^31^, whereas up to 0.44 mM wogonin did not exert any inhibitory effect against *C. albicans* (Fig. 3). Wogonin reportedly possesses antifungal activity against *Botrytis cinerea*, *Penicillium notatum*, *Penicillium frequentance*, and *C. neoformans*^32,33^. However, to the best of our knowledge, the mechanism of wogonin tolerance of *C. albicans* has never before been investigated in detail.

Efflux pumps, e.g., ATP-binding cassette pumps and major facilitator superfamily transporters, are transport proteins in the cell membrane. In *C. albicans* and other microbes, they are involved in the efflux of toxic substances to the extracellular milieu, contributing to cellular drug resistance^25–27,29^. One of the mechanisms of baicalein-induced apoptosis in *C. albicans* involves the inhibition of efflux pumps^28,31^. One possible explanation for the difference in antifungal activity spectra of baicalein and wogonin is that wogonin does not inhibit the efflux pumps of *C. albicans*, which ultimately results in protection from cell death or apoptosis. Another possibility is that differences in cell wall biosynthesis and chemical composition between *C. albicans* and dermatophytes^34–36^ lie at the basis of the ineffectiveness of the antifungal agent against *C. albicans* or at least enhance its resistance to the inhibitory effect. Previous studies have demonstrated that some antifungal compounds, such as *Juniperus* essential oil, *Syzygium aromaticum* essential oil, phlorotannins, geraniol, nerol, citral, neral, geranial, and psoriasin (redS100A7), are most effective against dermatophytes, or have no direct antifungal effects against *Candida* species^17,23,37–41^. Yeasts are hydrophilic and they lack hydrophobins; therefore, hydrophobic antifungal compounds accumulate at the fungal conidia^17,42–44^, which is another possible explanation for the difference in the antifungal activity spectra of baicalein and wogonin.

Flavonoids are phenolic substances that occur widely in the plant kingdom, with over 8,000 monomeric compounds identified to date. Because of their widespread ability to inhibit spore germination of plant pathogens, flavonoids have been suggested to be useful in the treatment of human infections by fungal pathogens^9^. For example, kaempferol has been considered a potential candidate against fluconazole-resistant *Candida* species because it inhibits the expression of cerebellar degeneration-related protein (CDR) 1, CDR2, and multidrug resistance protein 1^45^. Synergistic effects of baicalein and wogonin with available antifungal and antibacterial agents have been reported^30,31,46^. Importantly, baicalein and wogonin exert nearly no or minor cytotoxic effect on healthy human cells^47–50^. We therefore suggest that baicalein and wogonin are attractive candidates that might replace, or be used synergistically with, antimicrobial drugs, including anticancer drugs or immunosuppressive agents, to treat the ensuing fungal infections^51–53^.

In future, it will be necessary to test the antifungal activities of baicalein and wogonin against other pathomycetes and to evaluate the effects of baicalein and wogonin against fungal infections, e.g., tinea pedis, candidiasis, and aspergillosis, *in vivo*. Furthermore, investigation of the effects of baicalein and wogonin on fungal gene expression may improve our understanding of their structural differences, specificity, and antifungal mechanisms of action. Finally, further identification and enhancement of additional biological activities of baicalein and wogonin would expand their application potential.

## Conclusions

In summary, our findings suggest that baicalein and wogonin are major compounds with antifungal activity in Kampo medicine that elicit apoptosis-like programmed cell death in pathogenic fungi. The antifungal effects of baicalein and wogonin may lead to the development of new and safe treatment strategies, especially for the clinical treatment of infections caused by pathogenic filamentous fungi.

## Materials and Methods

### Fungal strains and isolates

*T. rubrum* (NBRC 5467), *T. mentagrophytes* (IFM 46027), *A. fumigatus* (NBRC 4400), and *C. albicans* (NBRC 1385) were obtained from the Culture Collection Division at the Biological Resource Center of the National Institute of Technology and Evaluation (Chiba, Japan). The fungi were cultured on Sabouraud dextrose agar containing chloramphenicol (50 μg/mL) (Nacalai Tesque, Kyoto, Japan) and cycloheximide (500 μg/mL) (Sigma-Aldrich, MO, USA) at 28 °C. After 7 days, conidia or yeast form were collected and stored at 5 × 10^6^ colony forming units/mL in an aqueous solution of 40% (v/v) glycerol at −80 °C^17^.

### Preparation of Ou-gon extracts

Ou-gon powder (standard commodity classification no. of Japan: 875100; product code: 5100019 × 1091) was provided by Tsumura & Co. (Tokyo, Japan). To prepare the extracts, 200 mg of Ou-gon powder was mixed with 1 mL of sterilised water, methanol (MeOH), ethanol (EtOH), or acetic acid in conical centrifuge tubes by vortex-mixing. The four extraction solutions were chosen based on previous studies that have demonstrated antifungal properties of different types of extracts^54,55^. The water extract was heated at 45 °C for 30 min in a Bi-515a block incubator (Astec, Fukuoka, Japan), and the mixtures were sonicated (Branson Sonifier 450; Emerson, Atsugi, Japan) for 5 min. All Ou-gon extracts were centrifuged at 22,140 × *g* at 4 °C for 15 min. The supernatants were sterilised by passing them through a 0.45 μM sterile syringe filter (Nihon Millipore, Tokyo, Japan). The solvents (MeOH, EtOH, and acetic acid) were removed by vacuum evaporation (Spin dryer light VC-36R; Taitec, Koshigaya, Japan), and the precipitates were reconstituted in 300 μL of 10% (v/v) dimethylformamide (DMF). The final concentration of the Ou-gon extracts in distilled water was 20 mg/mL. Baicalein (Cayman Chemical, MI, USA) and wogonin (Wako Pure Chemical Industries, Osaka, Japan) were dissolved in 10% (v/v) DMF because of low solubility in aqueous solutions and were diluted in distilled water at the desired concentration (final concentrations of baicalein were 0.46 mM, 0.23 mM, 0.12 mM, 0.06 mM, 0.03 mM, and 0.01 mM; final concentrations of wogonin were 0.44 mM, 0.22 mM, 0.11 mM, 0.06 mM, 0.03 mM, and 0.01 mM). The final DMF concentrations were 1% (v/v) in all assays. N,N,N′,N′-tetrakis(2-pyridylmethyl)ethylenediamine (TPEN) (Sigma), a cell-membrane–permeable metal ion Zn^2+^-chelator that inhibits the growth of fungi by inducing zinc depletion-induced apoptosis-like cell death, was used as a broad-spectrum antifungal agent^17^.

### *In-vitro* antifungal activity assay

The antifungal activity of each preparation was assayed using the microbroth dilution assay based on measuring the absorbance at 595 nm, and described in our previous reports^7,17^.

### Reversed-phase high-performance liquid chromatography

Ou-gon extracts were dissolved in 500 μL of 50% (v/v) MeOH and separated on a C18-RP-HPLC column (YMC-Triart C18, 150 × 4.6 mm i.d., S-5 μm, 12 nm; YMC, Kyoto, Japan) using AKTA Purifier system (GE Healthcare) with UV detection at 280 nm. MeOH in 0.04% phosphoric acid (46:54, v:v) was used as the mobile phase at a flow rate of 0.5 mL/min. All eluted fractions (2 mL) were collected by using the Frac-920 fraction-collector (GE Healthcare), and the mobile phase in all fractions was removed by vacuum evaporation. The precipitates were dissolved in 1% (v/v) DMF, and their activity was tested in *in-vitro* antifungal activity assays. Two authentic samples of baicalein or wogonin were dissolved in 50% (v/v) MeOH (final concentration 100 μg/mL) and analysed by RP-HPLC using the same approach.

### Liquid chromatography-electrospray ionization tandem mass spectrometry

Extract fractions displaying antifungal activity were analysed by LC-ESI-MS/MS. The analysis was conducted in a positive ion mode using a TSQ quantum mass spectrometer (Thermo Fisher Scientific, Tokyo, Japan). Flow injection analysis was used, with an isocratic mobile phase of 50% (v/v) MeOH in distilled water with 0.1% (v/v) formic acid. Mass peaks were selected and m/*z* values were calculated using Xcalibur 2.1 software (Thermo Fisher Scientific)^15^.

### SYTOX^®^ green uptake assay

*T. rubrum*, *T. mentagrophytes*, *A. fumigatus*, and *C. albicans* were cultured on Falcon 4-well culture slides for 36‒72 h, and then incubated for 24 h at 28 °C in Sabouraud liquid medium in the presence of either 0.46 mM baicalein or 0.44 mM wogonin^34^. SYTOX^®^ green (Molecular Probes-Invitrogen, OR, USA) was added at a final concentration of 1 μM. After 10 min of incubation with shaking in the dark, the fungal cells were mounted using a drop of fluorescence mounting medium (Dako, Glostrup, Denmark). Fluorescence was detected under an Olympus BX51 fluorescence microscope (Olympus, Tokyo, Japan) with an Olympus DP 21 digital camera system (excitation at 450–490 nm and emission at 523 nm).

### Terminal deoxynucleotidyl transferase dUTP nick end-labelling assay

The TUNEL assay was performed using an *in-situ* cell death detection kit (Fritz Hoffmann-La Roche, Basel, Switzerland). *T. rubrum*, *T. mentagrophytes*, *A. fumigatus*, and *C. albicans* were cultured on Falcon 4-well culture slides for 36‒72 h at 28 °C in Sabouraud liquid medium. The cells were then treated for 24 h with 0.46 mM baicalein or 0.44 mM wogonin, and the experiment was performed as described by Hein *et al*.^17^.

### Intracellular reactive oxygen species accumulation assay

The intracellular ROS accumulation assay was performed using *T. mentagrophytes* and *C. albicans*, the fluorescent dye 2′,7′-dichlorofluorescein diacetate, and a cellular ROS detection assay kit (ab113851; Abcam, MA, USA), following the manufacturer’s protocol. Fluorescence intensity was determined with a fluorescence microplate reader (DTX 880 Multimode Detector; Beckman Coulter, CA, USA).

### Mitochondrial membrane potential assay

The MMP assay was performed using *T. mentagrophytes* and *C. albicans*, the cationic dye 5,5′,6,6′-tetrachloro-1,1′,3,3′-tetraethylbenzimidazolylcarbocyanine iodide (JC-1), and an MMP assay kit (ab113850; Abcam), following the manufacturer’s protocol. Fluorescence intensity was determined with a fluorescence microplate reader.

### Scanning electron microscopy

Controls and baicalein-, wogonin-, or TPEN-treated *T. rubrum*, *T. mentagrophytes*, *A. fumigatus*, and *C. albicans* cells were grown on Sabouraud dextrose agar medium or in Sabouraud liquid medium for 24 h at 28 °C. The treated cells were collected in conical centrifuge tubes and fixed with 2.5% (v/v) glutaraldehyde in 0.1 M phosphate buffer (PB; pH 7.3) at 4 °C for 2 h. This was followed by five consecutive washes with 0.1 M PB, post-fixing with 1% (v/v) osmium tetroxide in 0.1 M PB at 4 °C for 2 h, and five consecutive washes with 0.1 M PB. The treated fungal cells were incubated with an aqueous solution of 1% (w/v) tannin (air-free) at 4 °C in the dark for 1 h and washed five times with 0.1 M PB. Samples were mixed with 1% (v/v) osmium tetroxide in 0.1 M PB x at 4 °C for 1 h and dehydrated in a graded EtOH series. The dehydrated samples were immersed five times in *t*-butyl alcohol and lyophilised by vacuum freeze-drying (JFD-320; JEOL, Tokyo, Japan). Samples were mounted using silver paste (Nisshin EM, Tokyo, Japan), sputter-coated with gold using an ion coater (VX-10A; Eiko, Tokyo, Japan), and monitored by SEM in the voltage range of 5–15 kV (JSM-6510; JEOL).

### Transmission electron microscopy

Baicalein-, wogonin-, and TPEN-treated *T. rubrum* cells grown in Sabouraud liquid medium were collected into conical centrifuge tubes and rinsed with 2.5% (v/v) glutaraldehyde in 0.1 M PB (pH 7.3) at 4 °C for 2 h. This was followed by washing with 0.1 M PB, post-fixing with 1.5% (w/v) potassium permanganate in distilled water at 4 °C for 16 h, and washing in chilled distilled water until the solution turned colourless. Then, the pellets were block-stained with 0.5% uranyl acetate in 50% (v/v) acetone at 4 °C for 1 h. Dehydration was achieved in a graded EtOH series using an automated routine tissue processor (Leica EM TP; Leica Microsystems K.K., Wetzlar, Germany), and the EtOH was replaced with *n*-butyl glycidyl ether. The samples were embedded in Epon resin (TAAB Epon 812 resin; TAAB Laboratories Equipment, Berks, UK), and ultrathin sections (60–80-nm thick) were prepared using an ultramicrotome (MT-7000; RMC, AZ, USA). The ultrathin sections were stained using uranyl acetate and lead citrate, placed on 300-mesh copper grids, and observed by TEM operated at 80 kV (JEM1200EX; JEOL)^56^.

### Statistical analysis

All data were obtained from at least three independent experiments and are expressed as the mean ± standard deviation (SD). Statistically significant differences between samples were evaluated using one-way analysis of variance followed by Dunnett’s post-hoc comparisons. The analyses were performed using SPSS V22.0, and *p* < 0.05 was considered significant.

## Data Availability

All data generated or analysed in this study are included in the published article.
